# Inequality in in-hospital mortality due to road traffic accident between ethnic populations in specified groups living in the same country

**DOI:** 10.1186/s13584-020-0363-z

**Published:** 2020-04-20

**Authors:** Abebe Tiruneh, Maya Siman-Tov, Irina Radomislensky, Israel Trauma Group, H. Bahouth, A. Becker, A. Hadary, I. Jeroukhimov, M. Karawani, B. Kessel, Y. Klein, G. Lin, O. Merin, B. Miklush, Y. Mnouskin, A. Rivkind, G. Shaked, G. Sibak, D. Soffer, M. Stein, M. Wais, H. Pharan, I. Garbetzev, Kobi Peleg

**Affiliations:** 1 Israel National Center for Trauma and Emergency Medicine, Gertner Institute for Epidemiology and Health Policy Research, Tel-Hashomer, Ramat Gan, Israel; 2 Department of Disaster Management, School of Public Health, Tel Aviv University, Tel Aviv, Israel

**Keywords:** Arabs, Jews, Road traffic injury, Mortality, Risk factor

## Abstract

**Background:**

Road traffic accidents (RTA) are not equally distributed between ethnic groups, disproportionately affecting minorities. In Israel, Arabs are at higher risk of involvement in RTA relative to their proportion in the population. This study aims to compare the risk of in-hospital mortality from RTA between Arabs and Jews in Israel and to identify the factors associated with mortality in each population group.

**Methods:**

This study is based on the Israeli National Trauma Registry of patients hospitalized due to road traffic injuries (Injury Severity Score 16+) between 2008 and 2017. Demographic, injury and hospitalization characteristics, evacuation means and in-hospital mortality were analyzed. Hierarchical multivariate logistic regression with random intercept for the treating hospital was performed to estimate the risk of mortality.

**Results:**

Of the 11,523 hospitalizations reported, 29% were Arabs, which is higher than their proportion in the Israeli population (21%). When comparing Arabs with Jews they were younger (ages 0–24 years - 61% vs 30%), injured as a car driver (28% vs 20%) or passenger (21% vs 15%) and less likely to be a motor cyclist (8.8% vs. 19.2%). In addition, Arabs were more likely to suffer from critical injuries (51% vs 44%) and head injuries (71% vs 66%). Although Arabs were less likely to be evacuated by ambulance (68% vs 80%), they were more likely to be evacuated by a private vehicle or an emergency medical helicopter. Transfers between hospitals were greater among Arabs (14% vs 22%), as were hospital admissions “outside official work hours” (70% vs 78%) and hospital resource utilization. After accounting for demographic, injury, and hospitalization characteristics the risk of in-hospital mortality was significantly higher among Arabs compared to Jews (OR: 1.63, 95% CI: 1.14–2.32). The significantly higher mortality among Arabs was apparent in the sub-group of patients who were critically injured and in those who arrived at the hospital “outside official work hours”.

**Conclusions:**

This study suggests the need for developing appropriate interventions focusing on the Arab community in general, and according to the analysis of risk groups and areas of injury in particular, including rapid access to emergency medical services and definitive care.

## Background

Globally, road traffic accidents (RTA) are a major cause of death, particularly among young people [1]. Global projections of mortality predict road traffic crashes to remain among the top ten leading causes of death by 2030 and by 2060, ranking 10th and 9th, respectively [2]. Prehospital treatment capability, rapid prehospital transport, and early hospital and surgical treatment capability are important factors for the survival of trauma casualties [3–6]. Road traffic accidents are not equally distributed between ethnic groups, with ethnic minorities typically bearing the greater share [1, 7, 8].

In Israel, for the last decade, there have been fluctuations in the number and rate of casualties and fatalities as a result of RTA [9–13], see Table 1. Between 2014 and 2017, there was a consecutive increase in the number of casualties from RTA (21,910 and 23,101 respectively for 2014 and 2017). While between 2014 and 2015 a 1.2% increase in the number of casualties was observed, a 3.9% increase took place between 2016 and 2017. The rate of casualties also increased between 2014 and 2017, from 264/100,000 residents to 268/100,000 residents, respectively [9]. The number of RTA-related fatalities steadily increased from 263 deaths in 2012 to 335 deaths in 2016, a 27.4% increase. Not only the numbers increased, but also the fatality rate, from 3.3/100,000 residents in 2012 to 4.0/100,000 residents in 2016 [9, 10]. There has also been a documented increase in the number of RTA-related hospitalizations, with a 10.1% increase from 8345 hospitalized RTA casualties in 2010 to 9190 in 2015 [14]. When comparing international injury rates with Israeli data, between 2013 and 2015, reported injury rates were higher in Israel than in some of the other countries, including Hungary, Greece, Norway, Poland, Finland and Sweden [15].

**Table 1:** RTA-related casualties, fatalities and hospitalizations, 2008–2017

Year	Casualties - CBS^a^		Fatalities - CBS^a^		Hospitalizations - INTR^b^	
	*n*	Rate per 100,000 residents	*n*	Rate per 100,000 residents	*n*	% change^c,d^
2008	31,811	434	412	5.6	–	–
2009	31,832	425	314	4.2	–	–
2010	28,084	368	352	4.6	8345	–
2011	27,141	349	341	4.4	8124	−2.6
2012	23,904	302	263	3.3	8305	+ 2.2
2013	24,294	299	277	3.4	8651	+ 4.2
2014	21,910	264	279	3.4	8462	−2.2
2015	22,164	262	322	3.8	9190	+ 8.6
2016	22,236	263	335	4.0	9365	+ 1.9
2017	23,101	268	321	3.7	9093	−2.9

*Abbreviations*: *RTA* road traffic accident, *n* number, % percent

^a^Based on the Central Bureau of Statistics data [9–13]

^b^Based on the Israel National Trauma Registry (INTR) database

^c^Percent change in the number of hospital admissions due to road traffic injuries in the specified year compared to the previous year

^d^In the year 2010 two hospitals were added to the trauma registry, so the data of the years 2008 and 2009 are not presented to enable the data to be comparable

There are two main ethnic populations in the Israeli society: the Jewish majority (74.6%) and the Arab minority (20.9%) in 2017 [16]. The Israeli Central Bureau of Statistics (CBS) data for 2014, 2016 and 2017 reported that Arabs had a greater proportion of road traffic injuries and fatalities relative to their proportion in the Israel society [9, 10, 17]. There are reports that Arabs have higher rates of unsafe road behaviors and noncompliance with traffic signs and regulations, including speeding, failure to maintain driving distance, making wrong turns, driving without using seatbelts, and not using child restraints [13, 18–21]. In addition, the road infrastructure in many Arab towns and villages is often considered to be of low quality, including narrow roads, lack of sidewalks and pedestrian crossings and visibility obstacles [13, 19, 20, 22]. In comparison to the general society, the Arab population in Israel is younger, has a delayed age of enrollment to preschool, has a larger family size, and has a lower socio-economic status (SES) [13, 16, 19, 22, 23]. All these factors may increase their risk of involvement in RTA, particularly in severe accidents that can lead to severe injuries and death [20, 21]. For example, young road users are more likely to engage in risky behaviors than their older counterparts, such as speeding, driving under the influence of alcohol, and not using helmets or seat belts, all of which may increase their risk of injury or death [24]. Low SES may be associated with less developed road infrastructure, risky road safety behaviors and use of vehicles that are not road worthy, all of which are reported to increase the risk of involvement in RTA [20, 25, 26].

The few available research studies on road traffic injury-related mortality between Arabs and Jews included different study populations and reported different results; while Magid et al. indicated increased mortality among Arabs [13], Abdel-Rahman et al. found no difference [20]. Uncovering the potential sources of disparity between ethnic groups may prove of great importance for future prevention and intervention efforts. The current study aimed to improve our understanding of this phenomenon by comparing in-hospital mortality following RTA between Arabs and Jews in Israel and identifying the risk and associated factors for in-hospital mortality in each population group.

## Methods

Data were drawn from the Israeli National Trauma Registry (INTR) database for the years 2008–2017. The INTR includes all hospitalized trauma patients classified with an International Classification of Diseases, Ninth Revision (ICD-9-CM) diagnosis code of 800–959.9; who were admitted to the Department of Emergency Medicine (ED) and hospitalized, died in the hospital (including deaths in the ED) or were transferred to or from another hospital. The INTR does not include casualties who died at the scene of the event or on the way to hospital; who were discharged following treatment in the ED; or who were admitted 72 h or more following the event. The registry does not include poisoning, drowning or choking. Data reported in the registry are recorded by trained trauma registrars at each trauma center under the supervision of a trauma director and trauma coordinator. Electronic files are transferred to the INTR at the National Center for Trauma and Emergency Medicine Research where quality assurance is carried out prior to data analysis. Unclear or erroneous data are referred back to the trauma centers for clarification or completion. The data in the INTR are anonymous and there is no way to identify patients. This study received the approval of the Sheba Medical Center Institutional Review Board (IRB) (Number 5138–18 SMC).

From this comprehensive database, patients with road traffic injuries were selected for this study. An Injury Severity Score (ISS) of 16+ was used as a criterion for inclusion because this has been shown to be associated with greater risk of mortality [15]. Data on transferred patients were abstracted from the records of the hospital they were transferred to in order to avoid double counting. Patients with unknown ethnicity (*n* = 11, 0.1%) and race other than Arab or Jews (*n* = 34, 0.3%) were excluded. As the study was meant to compare in-hospital mortality between Arabs and Jews of Israel; foreign workers (*n* = 2, 0.02%), tourists (*n* = 59, 0.5%) and Arab residents of East Jerusalem (*n* = 442, 3.7%) who are not Israeli citizens were excluded. The final cohort comprised 11,523 hospitalized patients; 29.3% were Arabs and 70.7% were Jews.

The data included demographic variables, injury characteristics, means of evacuation, hospitalization characteristics and in-hospital mortality. Injury Severity Scores were classified into three groups: 16–24, 25–49 and 50–75 (based on the recommendations for classification of severely injured patients) [27]. Injured body regions were grouped into five regions based on the Barell Injury Diagnosis Matrix: head and neck, spine, torso, extremities, and system-wide or unspecified [28]. Number of injured body regions was categorized as single (when only one body region was injured) or multiple (more than one injured body regions). Traumatic brain injury (TBI) was categorized as Isolated TBI or TBI+. Isolated TBI was defined as TBI-only or TBI plus injury to other body region (s) with Abbreviated Injury Score (AIS) less than 3; while TBI+ was defined as TBI plus injury to other body region (s) with AIS 3–6. Weekday was defined as working day hours from 08:00 to 16:00 Sunday through Thursday (in Israel Sunday is a work day); while weeknight was defined as night hours from 16:01 to 07:59 for Sunday to Thursday; and weekend was defined as 16:01 Thursday through 07:59 Sunday. The term “Outside official work hours” was used in this study to refer to weeknight and weekend combined. Annual injury admission rates due to RTA were calculated based on data from the National Trauma Registry and population data from the CBS [16].

Statistical analyses were performed using SAS statistical software version 9.4 (SAS Institute, Cary, NC, USA). Descriptive data were compared using χ^2^-test, and a hierarchical multivariate logistic regression model with random intercept for the treating hospital was performed to estimate the risk of mortality among Arabs compared with Jews. Within the model, the effect estimate of the population group (Arabs vs. Jews) was determined with adjustment for multiplicity using Bonferroni correction for injury severity categories (ISS 50–75, ISS 25–49, ISS 16–24), for patient means of evacuation (ambulance emergency medical services (EMS), helicopter EMS), and for patient time of hospital arrival (“Outside official work hours”, weekday). The model accounted for age (continuous), gender, ISS, type of injury (penetrating vs. non-penetrating), means of evacuation, whether patient was transferred from another hospital or arrived directly from scene, time of hospital arrival, undergoing diagnostic imaging in ED: computerized tomography scan, magnetic resonance imaging, ultrasound or X-ray examination,(yes/no), hospital length of stay (< 7 days versus > 7 days), admission to intensive care unit (yes/no), undergoing surgery (yes/no), with random intercept for the treating hospital. Descriptive data values are reported as percentages, and estimates of the logistic regression model are expressed as odds ratio with 95% confidence interval. A *p*-value of < 0.05 was considered statistically significant.

## Results

### Demographic and injury characteristics

The proportion of Arabs hospitalized due to RTA (29.3%) was higher than their share in the general Israeli population (20.9%), while for Jews it was lower (70.7% vs. 74.6%). Annual trauma hospital admission rates due to RTA among Arabs was greater compared with that of Jews for each year during the study period. For example, in 2017 the trauma admission rate among Arabs was 21.6/100,000 population compared with 12.6/100,000 population among Jews. Similarly, in 2008, Arabs had an admission rate of 20.3/100,000 population compared with 14.2/100,000 population among Jews. In comparison to Jews, Arabs were younger; 60.9% of hospitalized traffic casualties among Arabs were ages 0–24 years compared to 29.6% among Jews. Males constituted the vast majority of road traffic casualties in both ethnic groups, with the proportion being greater among Arabs (81.2 and 70.7%, respectively for Arabs and Jews).

As displayed in Table 2, Arabs were more likely to be injured as a car occupant while Jews were more likely to suffer from motorcycle and bicycle-related injuries. Arabs had significantly higher rate of critical injuries (ISS 25+). For each type of road user, except bicyclists, Arabs, in comparison to Jews, were at greater risk for sustaining critical injuries, with the most striking difference being among car passengers (53.5% versus 44.0%, *p* < 0.001), car drivers (51.6% versus 42.7%, *p* < 0.0001) and motorcyclists (54.9% versus 46.6%, *p* < 0.05) (data not shown).

**Table 2:** Hospitalizations due to road traffic injuries (ISS 16+): Demographic and injury characteristics by ethnic group

Variable ^a,b^	Total (N = 11,523, 100%)	Arabs (*n* = 3372, 29.3%)	Jews (*n* = 8151, 70.7%)	*p*-value^c^
	%	%	%	
Age (year)				*p* < 0.0001
0–14	14.2	25.5	9.6	
15–24	24.5	35.4	20.0	
25–44	24.5	22.9	25.1	
45–64	19.4	10.6	23.1	
65+	17.4	5.6	22.3	
Gender				*p* < 0.0001
Male	73.7	81.2	70.7	
Type of road user				*p* < 0.0001
Car Driver	22.7	28.2	20.4	
Car passenger	16.9	21.4	15.1	
Motorcyclist	16.2	8.8	19.2	
Bicycle rider	11.2	9.1	12.1	
Pedestrian	27.6	26.4	28.1	
Undefined transport category	0.8	1.3	0.6	
Other road users^d^	4.6	4.8	4.4	
Injury type				*P* = 0.0004
Penetrating	0.6	1.0	0.5	
ISS				*p* < 0.0001
16–24	54.3	49.5	56.2	
25–49	41.0	45.0	39.3	
50–75	4.8	5.5	4.5	
Injured body region				
Head and neck (yes)	67.3	70.9	65.8	p < 0.0001
Spine and back (yes)	30.8	30.3	31.0	*P* = 0.5017
Torso (yes)	73.8	75.8	72.9	*P* = 0.0012
Extremities (yes)	52.1	52.3	52.1	*P* = 0.7788
System-wide/Unspecified (yes)	1.8	2.2	1.6	*P* = 0.0231
Number of injured body region(s)				*p* < 0.0001
Single	25.6	23.0	26.7	
Multiple	74.4	77.0	73.3	
TBI				*P* < 0.0001
Isolated TBI	14.6	12.8	15.4	
TBI+	33.2	35.6	32.2	
No TBI	52.2	51.6	52.5	

^a^*Abbreviations*: *ISS* injury severity score, *AIS* abbreviated injury scale, *TBI* traumatic brain injury

^b^There were no missing data for the variables displayed except for gender 2 (0.017%) and injury type 1 (0.009%),

^c^Determined using *x*^*2*^*-tests*

^d^Others included users of all-terrain vehicles, scooters, animal riders

The comparison groups also differed in the type of injured body region, with injuries to head, torso injuries or multiple injuries being significantly more common among Arabs. Arabs were more likely to have a higher rate of TBI+ (Table 2).

### Hospitalization characteristics and evacuation means

While Jews were more likely to be evacuated to the hospital by ambulance (79.5 and 68.0%, respectively for Jews and Arabs), evacuation by private vehicle and EMS helicopter were more common among Arabs (Table 3). Transfers between hospitals, hospital admissions “outside official work hours”, and hospital resource utilization were greater among the Arab population. Among Arabs, 21.8% were transferred compared to 14.2% among Jews; 77.6% of Arabs were admitted “outside official work hours” compared to 70.0% of Jews (*p* < 0.0001); 79.9% of Arabs were treated in the trauma resuscitation unit, compared to 69.8% among Jews; 61.0% were admitted to the intensive care unit (ICU) compared to 51.4% among Jews; and 51.7% underwent surgery compared to 44.8% among Jews. However, there was no significant difference between the groups in length of hospital stay (LOS) or in type of treating hospital (Table 3).

**Table 3:** Hospitalizations due to road traffic injuries (ISS 16+): Hospitalization characteristics and in-hospital mortality by ethnic group

Variable ^ab^	Total (*N* = 11,523, 100%)	Arabs (*n* = 3372, 29.3%)	Jews (*n* = 8151, 70.7%)	*p*-value
	%	%	%	
Means of evacuation				*p* < 0.0001
Ambulance EMS	76.1	68.0	79.5	
Helicopter EMS	7.1	11.2	5.5	
Private car/others	16.7	21.8	14.2	
Transferred from another hospital (yes)	16.4	21.8	14.2	*P* < 0.0001
Patient arrival time at hospital				*P* < 0.0001
Weekday	27.8	22.4	30.0	
Weeknight	33.0	34.8	32.3	
Weekend	39.2	42.8	37.7	
Treated in trauma resuscitation unit (yes)	72.7	79.9	69.8	*P* < 0.0001
ICU admission (yes)	54.2	61.0	51.4	*p* < 0.0001
Undergoing surgery (yes)	46.8	51.7	44.8	*p* < 0.0001
Hospital LOS for 7 days or more (yes)	58.5	58.7	58.5	*P* = 0.8306
Hospital LOS for 14 days or more (yes)	33.4	33.5	33.3	*P* = 0.7708
CT MRI US X-ray performed in ED (yes)	94.3	94.6	94.1	*P* = 0.3328
Hospital type Level I trauma center	75.0	76.0	74.5	*P* = 0.0818
Level II trauma center	25.0	24.0	25.5	
In-hospital mortality (yes)	8.7	8.2	8.9	*P* = 0.2427

^a^*Abbreviations*: *EMS* emergency medical service, *ICU* intensive care unit, *LOS* length of stay, CT MRI US X-ray-computed tomography scan or magnetic resonance imaging or ultrasound or X-ray examination combined together, ED-emergency department

^b^There were no missing data for the variables presented except for transfer from another hospital, hospital arrival time and LOS for 7 days or more, which had missing values for 25 (0.217%), 3 (0.026%) and 2 (0.017%) observations, respectively

### In-hospital mortality

Of the hospitalized patients with severe and critical injuries (ISS16+), 8.7% died in the hospital with no significant differences between Arabs and Jews. However, Arabs and Jews are significantly different in demographic, injury and treatment-related characteristics relevant to impact on mortality. After adjusting for age, gender, ISS, type of injury, means of evacuation, transfer status, time of hospital arrival, undergoing diagnostic imaging in ED, LOS, admission to ICU and undergoing surgery with random intercept for the treating hospital, the risk of mortality was 1.6 times greater among Arabs compared to Jews (OR: 1.63, 95% CI: 1.14–2.32, *p* < 0.01), see Table 4. Significantly higher mortality among Arabs compared to Jews was apparent among patients who were critically injured [ISS 50–75:- OR:1.73, 95% CI: 1.09–2.75, *p* < 0.01; and ISS 25–49:- OR:2.10, 95% CI: 1.03–4.25, *p* < 0.05], and among patients who were admitted “outside official work hours” (OR: 1.68, 95% CI: 1.04–2.73), see Table 4.

**Table 4:** Risk of in-hospital mortality due to road traffic injuries (ISS 16+) among Arabs compared with Jews

Group^a^	OR (95% CI)	AUROC
Overall^b^	1.63 (1.14–2.32)**	0.898
ISS50-75^c^	1.73 (1.09–2.75)**	
ISS25–49 ^c^	2.10 (1.03–4.27)*	
ISS16–24 ^c^	1.19 (0.53–2.68)	
Ambulance EMS ^c^	1.32 (0.87–1.99)	
Helicopter EMS ^c^	1.33 (0.60–2.95)	
“Outside official work hours”^c^	1.68 (1.04–2.73)*	
Weekday ^c^	1.57 (0.83–3.00)	

*Abbreviations*: *ISS* injury severity score, *EMS* emergency medical service, *OR* odds ratio, *CI* confidence interval, *AUROC* area under the receiver operating curve

*p < 0.05, **p < 0.01

^a^The model was adjusted for age (continuous), gender, ISS (16–24, 25–49 and 50–75), type of injury (penetrating versus non-penetrating), means of evacuation (ambulance EMS, helicopter EMS and Other), whether patient was transferred from another hospital or directly arrived from scene, time of hospital arrival (“outside official work hours”, weekday), undergoing diagnostic imaging in emergency department: computed tomography scan, magnetic resonance imaging, ultrasound or X-ray examination (yes/no), hospital length of stay (< 7 days versus > 7 days), admission to intensive care unit (yes/no), undergoing surgery (yes/no) with random intercept for the treating hospital

^b^Overall was to describe the entire study population

^c^Within the same model, effect estimate of Arabs versus Jews was determined with adjustment for multiplicity using Bonferroni correction

A sensitivity analysis was performed to estimate in-hospital mortality by entering into our model pre-hospital time, excluding and incorporating its missing data, assuming this variable is a significant predictor of survival. There was a high missing rate with regard to hour of injury (36.6% for the overall study population, 33.8% for Arabs and 37.8% for Jews). Similar results were observed in both models to that of the final model reported in this study. Nevertheless, we opted to exclude pre-hospital time in our final model because of its high missing data rate. Importantly, our final model, that excluded prehospital time, had a good performance (Area Under Receiver Operating Characteristic = 0.898). Moreover, in order to comprehend whether this high AUROC was not a result of overfitting, we tested our model using a new set of data for the year 2018 where the concordance index was observed to be 0.9, which would support the reliability of our data.

## Discussion

The study outcomes indicate that there is no significant difference in the crude mortality due to RTA between Arabs and Jews. However, after accounting for possible confounding factors, mortality among Arab casualties was greater in comparison to Jewish casualties. The fact that this difference emerges after adjusting for confounders can be explained by the differential distributions and imbalance between Arabs and Jews in risk factors for mortality. The favorable characteristics among Arab casualties could exert a stronger effect in masking the mortality difference expected that would arise from the risk factors. Firstly, Arab casualties were younger than their Jewish counterparts, which was noticeable even after taking into consideration their younger age in the general population. In 2017, for example, the percentage of persons aged 0–24 years was 54.5% in the Arab population vs. 41.2% in the Jews population [16, 23]. This could mask the true mortality difference in the unadjusted mortality, as supported by our observation in the adjusted analysis. Secondly, additional hospitalization characteristics could be considered favorable to Arab casualties in masking the expected mortality difference between Arabs and Jews. For example, there were significant differences in hospital resource utilization, which is very likely to stem from the fact that Arab casualties suffered from more severe injuries compared with Jews. Arabs had greater rates of utilization of high impact interventions such as treatment in trauma resuscitation unit, surgical intervention and admission to the ICU, as well as evacuation by helicopter EMS; all of which would be expected to properly address the possible risk factors for increased mortality. This is supported by our observation that the difference in mortality between Arabs and Jews became apparent when hospitalization characteristics (hospital admission time, undergoing imaging study, undergoing surgery, admission to ICU, LOS) and means of evacuation were entered into a model accounting for age, gender, ISS, injury type and transfer status. This implies that hospitalization characteristics and means evacuation were favorable to Arabs to be discharged alive from hospitalization following RTA. Rapid prehospital transport, early hospital and surgical treatment are among the critical factors to improve trauma outcomes [3–6]. The differential rates of health care interventions between the comparison groups could reflect the appropriateness of the practice of trauma care in the country, i.e., treatment is received according to needs, independently of population groups, without any discrimination. To that end, the findings of this study confirm not only the efficacy of the Israeli trauma care system but also reinforce that the national health insurance law provides the necessary medical services to all residents in an effort to narrow the gaps of inequality between population groups [29–31].

Critical (ISS 25+) and multiple injuries (especially including TBI) not only characterize a large proportion of the injuries among Arab casualties, but also increase the risk of mortality. This is supported by the significant reduction in the odds ratio when ISS or TBI were included into the model thus influencing mortality outcomes. It is well established that injury severity is a strong predictor of mortality, with a strong inverse relationship between injury severity and mortality [27, 32]. Likewise, severe TBI is a major risk factor for mortality [33–35]. The differences in injury characteristics between Arabs and Jews can be presumed to be related to their differences in road safety behavior such as seatbelt use, helmet use, use of child restraints, compliance with traffic rules and signs, as well as road infrastructure differences [13, 19–22]. In addition, the severity of crashes and its consequences on mortality outcomes may be somewhat related to travel distances (for work and education) from the periphery and rural areas to major cities where most of the employment, universities and major business centers are found [13]. Long travel distances increase exposure to RTA due to driving behaviors, especially speeding and fatigue, which increase the risk of severe and fatal accidents. This is supported by the fact that in Israel 54% of persons killed in road accidents and approximately 34% of those seriously injured occurred on non-urban roads [21]. There is also evidence to suggest that the risk of dying in a road traffic crash still depends, in great part, on where people live and how they travel [1]. Time of arrival at the hospital may be another factor contributing to the disparity in mortality between Jews and Arabs. In this study, Arabs were more likely to be admitted “outside official work hours”, which has been associated with a greater risk of mortality [36]. Although this study did not investigate factors related to “outside official work hours”, it is suggested that optimal level of care may be hampered “outside official work hours” due to reduced staffing, fatigue, non-availability of senior staff, reduced resources and use of diagnostic procedures and interventions. Health professionals working “outside official work hours” may be less experienced and have less seniority, which can ultimately affect the outcome of care among severe and critical casualties. “Outside official work hours” not only there may be fewer supervisors but also the available supervisors may be required to overseeing the work of staff members they do not know well [35, 37].

Specific subgroups of Arab casualties were found to be at higher risk for in-hospital mortality following RTA, in comparison to Jews; including, patients suffering from critical injuries (ISS 25+) and those admitted “outside official work hours”. No significant difference was found between Arabs and Jews who were admitted on weekdays. A large proportion of Arab Israelis reside in the periphery and in rural areas - along with scattered villages, which are geographically far from the major cities where hospitals, particularly level I trauma centers are located [19, 20, 22, 38]. Occurrence of injury due to RTA in such areas may lead to prolonged prehospital times due to longer distances and travel times from the accident sites to the trauma centers, and thus contributing to increased mortality rates [5]. Among patients who were admitted “outside official work hours”, when optimal level of care may be hampered [35–37], the impact of distance and travel time on mortality outcome may be even stronger for patients with severe and critical injuries, TBI, or injuries involving multiple body regions, which all are more prevalent among Arabs compared with Jews. The differences in mortality between Arab and Jewish causalities (after adjusting for the confounders, including prehospital time) can be attributed, at least in part, to ethnicity per se or to unknown factors that this study was not be able to identify.

This study is based on data from trauma centers throughout the country, providing a vast geographical coverage and nationally representative data of hospitalized trauma casualties. In an effort to minimize mortality disparities between Jewish and Arab traffic casualties, future studies should collect more detailed information, such as travel distances between the scene of the event and where the definitive care is received.

### Limitations

The first limitation stems from the inclusion parameters of the INTR, which is the source of data for this study. Since the registry does not include patients who died at the scene of the event or on the way to hospital, or those patients who were not hospitalized; this study lacks both minor injuries and fatalities prior to arriving at the hospital. While these two extremes might differ between Arab and Jewish casualties, this study only included severe and critical injuries (ISS 16+) and thus the lack of minor injuries in the trauma registry does not affect this study. Although fatalities outside the hospital are not included, all severe and critical casualties receiving definitive care at one of the many trauma centers in Israel should be included in the registry, providing plentiful, representative and valuable information in understanding injury characteristics and outcomes, and specifically regarding injury-related hospitalizations.

Another limitation could be that co-morbidity, which affects mortality outcome, was not taken into account. However, co-morbidity was unlikely to affect our findings because the increased mortality among Arabs versus Jews persisted even after excluding from the analysis patients aged 65 years or above, among whom outcome after injury is more likely to be influenced by preexisting conditions [39]. In addition, due to lack of data this study did not examine the effect of other confounding factors, such as complications, serum lactate level and base deficit; which are associated with mortality [40–42]. On the other hand, ISS, a widely used tool for assessing injury severity and for determining outcome in trauma patients, was taken into account in the model [27, 43, 44]. While the most important limitation of ISS is with regard to penetrating trauma [45, 46], in this study the majority of casualties suffered from non-penetrating trauma, thus, the mortality predicting ability of ISS was unlikely to be affected.

Further, this study did not investigate the influence of socioeconomic variables and road safety practices, which could partly explain some of the results. Exploring those factors in future studies may further strengthen the outcome of this study which took into account a large number of covariates. There is evidence suggesting that characteristics, such as unsafe road practices and lifestyles, are related to increased risk of severe injury and mortality [13, 19, 20, 22, 47, 48].

## Conclusions

In comparison to Jews, in-hospital mortality due to RTA was greater among Arabs, particularly those who suffered from critical injuries and those admitted “outside official work hours”. It can be speculated that there may be synergistic interaction effects between more severe injuries, hospital arrival time “outside official work hours” and possibly increased prehospital time due to longer distances from injury scene to trauma center, which may increase the risk of mortality among Arab casualties. The outcomes of this study indicate the need for developing appropriate interventions focusing on the Arab community in general, and according to the analysis of risk groups and areas of injury in particular, including rapid access to EMS and definitive care.

### Policy implications

The outcome of this study identifies RTA as a source of inequality for in-hospital mortality between Arabs and Jews in Israel, which was particularly apparent among casualties who suffered critical injuries (ISS 25+) and in those who arrived in hospital “outside official work hours”. Building on our assessment, we propose that appropriate road traffic injury prevention and intervention programs be developed and implemented focusing on the Arab community in general, and according to the analysis of risk groups and areas of injury in particular in accordance with the identified characteristics.

Israel has adopted and implemented a series of road safety policies and strategies, with the Israel National Road Safety Authority being a lead agency. Enforcing traffic laws such as speeding, driving under the influence of alcohol, use of safety belts and child restraints, use of motorcycle helmet and mobile phone use have had varying results to date [1]. Although national laws, action plans, policies and programs are available in the country, their enforcement and implementation may not be adequate on levels necessary to achieve reductions in RTA inequalities, and consequently on mortality. In the wake of our results and in an effort to reduce inequalities, policy makers should use the data to appropriately allocate financial, material and human resources to promote RTA prevention, response and research. Governments need to take action to address road safety in a holistic manner. This requires involvement from multiple sectors such as transport, police, health, education, and actions that address the safety of roads, vehicles, and road users [49, 50]. The global plan for the decade of action for road safety 2011–2020 promotes proven, cost-effective solutions to improve road safety including those pertaining to road safety management, safer roads and mobility, safer vehicles, making road users safer, and improved post-crash response and hospital care [49].

Finally, hospitals and EMS need to anticipate ethnic variation in injury severity and hospitalization characteristics so as to optimize patient outcome and operational efficiency following RTA. These trauma care organizations can play a crucial role in both survival and disability outcomes following injury. Data reporting, research and evaluation should be an integral part of each medical provider in order to optimize patient care and outcomes. Trauma care organizations can contribute to RTA prevention by helping identify risk factors and circumstances via sharing anonymous data with relevant stakeholders. Serving as educators, trainers, consultants, advocates or coordinators in joint community-based activities; and actively participating with ministry of health in data reporting, research and evaluation health professionals can help lower the rate of RTA.

### What the present study adds to existing knowledge?

 ○ Overall, in comparison with Jews, Arabs have a higher risk of in-hospital mortality following RTA.

○ The higher mortality among Arabs versus Jews was apparent in sub-group of patients who were critically injured and in patients who arrived in hospital “outside official work hours”.

○ There is a vital need to formulate and implement population-specific and socio-culturally appropriate RTA prevention and response programs.

## Data Availability

The datasets generated and analyzed during the current study are not publicly available due hospitalization privacy but are available from the corresponding author on reasonable request.
